# Necrosis of the Palate and Superior Maxillary Bones, the Result of Alveolar Abscess and a Rubber Plate

**Published:** 1889-08

**Authors:** Wm. D. Babcock

**Affiliations:** Los Angeles, California


### NECROSIS OF THE PALATE AND SUPERIOR MAXILLARY BONES, THE RESULT OF ALVEOLAR ABSCESS AND A RUBBER PLATE.[1]

BY WM. D. BABCOCK, M.D., LOS ANGELES, CALIFORNIA.

THE patient was Miss X., a seamstress, aged twenty-seven, and of general good health. The only exception was her menstruation, which was irregular, six months sometimes elapsing between the periods. The family history was excellent.

At fourteen she first began to have trouble with her teeth, the pain usually being on the right upper side. All the teeth on this side were affected, and at times those on the left. The two lower molars on the right side had also given her trouble.

These attacks continued intermittingly until five years ago, when she began to have abscesses at the roots of the teeth. She frequently sought the aid of a dentist; but at last, in order to obtain

[1] Read before the third annual meeting of the Southern California Odontological Society.

relief, had all the upper teeth extracted. Before these were pulled nearly every one was discharging offensive pus. She now had a rubber plate fitted. In May, 1885, she noticed that her nasal catarrh, which had previously been slight, was rapidly growing worse. Her head, too, gave her considerable annoyance, feeling as if it were stopped up by a bad cold. After a season of apparent recovery, she had, in June, 1886, a return of the catarrhal symptoms, and has suffered from these more or less ever since.

In September, 1887, she first noticed an opening in the hard palate, at the junction of the superior maxilla and the palate bone, though she had never felt any particular tenderness in or sponginess of the hard palate.

I saw her first in November, 1887, when I removed the vomer; and since then I have also removed a portion of the palate processes of both palate and superior maxillary bones, along with a part of the alveolar process of the right maxilla, between the cuspid and bicuspid teeth.

She has now in the hard palate an oval opening three-fourths of an inch long and half an inch in width at the widest part. I had never before seen such rapid and wholesale destruction of bone, unless it was caused by syphilis; yet, though I have inquired most carefully into the case, telling her of my suspicions, I have been able to find no sign of syphilis. She willingly submitted to a course of antisyphilitic treatment, but without the slightest beneficial effect.

The woman, when she came to me, was almost distracted, fearing that she had this disease, since her family physician had said that such bone destruction could come from syphilis only. The only exposure she ever could have had was from a young woman, a room-mate, who slept with her, and who had some trouble with her genitals, the nature of which my patient could not learn.

After finding that the treatment did no good, I began to look about for other possible causes.

I noticed that although she was wearing a well-made rubber plate, yet the gums were red, swollen, and tender throughout the extent covered by the plate, and I advised replacing it with a metal plate. Since wearing this she has made a rapid change for the better. The odor and the discharge ceased; the gums became hard and of a natural color, and the destruction of bone entirely stopped.

This case was to me an unique and instructive one. The trouble was first an alveolar abscess, and then destruction of the periosteum.

The process now following up the lymph canals, invaded the Schneiderian mucous membrane, on account of its intimate connection with the previously affected parts, and gave rise to the so-called catarrh. The bone destruction was aided, in my opinion, by the rubber plate; for as this is a non-conductor of heat, it would prevent—the mischief once started—the heat escaping from the point of abscess formation, and thus increase the trouble.

